# Complete Genome Sequence of the Novel *Roseimicrobium* sp. Strain ORNL1, a Verrucomicrobium Isolated from the Populus deltoides Rhizosphere

**DOI:** 10.1128/MRA.00617-20

**Published:** 2020-07-02

**Authors:** Mircea Podar, Joel Turner, Leah H. Burdick, Dale A. Pelletier

**Affiliations:** a Biosciences Division, Oak Ridge National Laboratory, Oak Ridge, Tennessee, USA; DOE Joint Genome Institute

## Abstract

*Roseimicrobium* sp. strain ORNL1 is a soil bacterium that belongs to the phylum *Verrucomicrobia* and was isolated from the rhizosphere of a forest Eastern cottonwood tree, Populus deltoides, in Tennessee. Its 7.9-Mb chromosome was completely sequenced using PacBio long reads and is predicted to encode 6,288 proteins and 76 RNAs.

## ANNOUNCEMENT

*Verrucomicrobia* are bacteria found in many aquatic and terrestrial environments; some are symbionts of animals, but relatively few have been cultured (1). In soils, they have been found associated with plant roots, being enriched in the rhizosphere, and using exudates as a carbon source (2–4). Soil *Verrucomicrobia* are oligotrophs, and their abundance decreases upon nitrogen fertilization but increases after deforestation, when emerging grass exudates stimulate their growth (5, 6).

We present here the complete genome sequence of *Roseimicrobium* sp. strain ORNL1, isolated from the rhizosphere of a mature forest Populus deltoides in Oak Ridge, Tennessee (lat 35^°^55′18″N, long 84^°^10′25″W). A root-associated soil sample was used to obtain a microbial fraction by centrifugation on Histodenz medium (7). Single cells were randomly deposited by flow cytometry sorting (7, 8) on agar-containing DSMZ medium 1426 and incubated at 28°C to form colonies. Taxonomic characterization of individual colonies was performed by amplification of the small-subunit (SSU) rRNA gene using the universal primers 25F and 1492R (9), followed by Sanger sequencing. Based on ClustalW v2.1 sequence alignment, the rRNA gene amplified from a pink bacterial colony had a 99% pairwise identity with that of Roseimicrobium gellanilyticum, a species of *Verrucomicrobia* isolated from a soil consortium in Japan (10). Therefore, we designated our isolate *Roseimicrobium* sp. strain ORNL1.

A culture of *Roseimicrobium* sp. strain ORNL1 was grown in liquid R2A medium for 3 days at 30°C. Genomic DNA was extracted and purified using a Qiagen DNeasy kit and a Zymo Research DNA Clean & Concentrator kit, followed by shearing with Covaris g-TUBEs (Woburn, MA) to an average fragment size of 10 kb. A library was prepared with SMRTbell template prep kit v1.0 (Pacific Biosciences, Menlo Park, CA) and sequenced on a Pacific Biosciences Sequel instrument. Sequence quality-based filtering and assembly were performed using the software HGAP4 implemented in the PacBio SMRTLink v8.0 pipeline, using a target genome size of 8 Mbp (based on the Roseimicrobium gellanilyticum genome), a minimum concordance of 70, a seed coverage of 30-fold, and default settings for the other options. In all, 67,880 filtered subreads (*N*_50_, 9,123 nucleotides [nt]) from 381,770 total subreads were assembled into a polished contig 7,957,557 nt long, with 89% mean concordance, 193-fold mean coverage, and a G+C content of 59.9%. Using Geneious v11 (11), we mapped reads to both ends of the contigs and identified a common overlap that resulted in a circular chromosome of 7,957,748 bp. Gene prediction and functional annotation were performed using the NCBI Prokaryotic Genome Annotation Pipeline (PGAP) v4.8 (12), which identified 6,288 protein coding sequences, 67 tRNAs, 2 rRNA operons, and 3 noncoding RNAs (ncRNAs). A metabolic model was generated using KBase (13) and is accessible at https://narrative.kbase.us/narrative/56021, together with a RAST annotation. The average nucleotide identity (ANI) relative to Roseimicrobium gellanilyticum was calculated with FastANI v0.1.2 (14), implemented in KBase. The resulting ANI value of 84% suggests that *Roseimicrobium* sp. strain ORNL1 may represent a novel species.

### Data availability.

The *Roseimicrobium* sp. strain ORNL1 genome sequence has been deposited in GenBank under the accession number CP049143. The version described in this paper is the first version, CP049143.1. The PacBio reads have been deposited in the SRA under the accession number SRR11248338.
